# A service evaluation of PTSD Resolution therapy for military veterans

**DOI:** 10.1093/occmed/kqaf012

**Published:** 2025-03-31

**Authors:** C E Hall, N Greenberg

**Affiliations:** Department of Psychological Medicine, King’s College London, London SE5 9RJ, UK; Department of Psychological Medicine, King’s College London, London SE5 9RJ, UK

## Abstract

**Background:**

Post-traumatic stress disorder (PTSD) Resolution is a UK-based charity that provides treatment for military veterans, reservists and their families. However, there is little contemporary evaluation of their clinical outcomes to inform commissioners or potential service users.

**Aims:**

To establish whether treatment by PTSD Resolution therapists resulted in positive outcomes at the end of therapy and follow-up; to establish risk and resilience factors associated with positive treatment outcomes; and to the extent possible, compare PTSD Resolution with National Health Service (NHS)—Improving Access to Psychological Therapies (IAPT) services.

**Methods:**

A sample of 211 closed cases from the service provider between April 2022 and May 2023 were utilized. Clients provided demographic data and completed a series of mental health screening tools. Follow-up data were also collected where possible. Paired *t*-tests, univariable binary logistic regressions and chi-squared tests were used in the analysis.

**Results:**

Around 6% of clients attended only one session, with 82% having a planned ending. This service evaluation suggests that veterans who enter therapy with PTSD Resolution appear to experience similar rates of recovery to IAPT users. Analysis of follow-up data revealed that clients scores slightly increased following the completion of therapy but remained below caseness thresholds and significantly lower than entry-level scores.

**Conclusions:**

These data suggest that veterans who choose to engage with PTSD Resolution for their mental health difficulties should expect to experience a similar benefit to that they would have experienced if they had sought outpatient care from the NHS.

## Teaser

Post-traumatic stress disorder (PTSD) Resolution, a UK charity providing support for military veterans, provided 211 closed cases to King’s College London for independent evaluation of their services. Results indicated that clients reached recovery rates similar to National Health Service (NHS) ‘Improving Access to Psychological Therapies’ users. Follow-up scores remained below clinical thresholds post-therapy. In conclusion, veterans receiving mental health support from PTSD Resolution should expect to experience similar benefits to those they would experience from NHS outpatient care.

**Key learning points**

**What is already known about this subject:**

- In 2019, Burdett and Greenberg published the King’s Centre for Military Health research evaluation of PTSD Resolution, a UK-based charity that provides treatment for military veterans, reservists and their families.
- It was concluded that the services PTSD Resolution appears to be an acceptable alternative to treatment offered through the National Health Service but avenues for future evaluation remained.

**What this study adds:**

- Following a real-world evidence approach, this service evaluation provides a contemporary assessment of PTSD Resolution’s clinical outcomes.
- Data suggests PTSD Resolution clients appear to experience similar rates of recovery to National Health Service Improving Access to Psychological Therapies users.

**What impact this may have on practice or policy:**

- The evaluation suggests that veterans, and their family members, who choose to engage with PTSD Resolution for their mental health difficulties should expect to experience a similar benefit to that they would have experienced if they had sought outpatient care from the National Health Service.

## Introduction

The UK government has committed the nation to preventing disadvantage in access to public and commercial services for the Armed Forces Community through the Armed Forces Covenant, introduced in 2011 [1]. Additional veterans-focused commitments include the 2018 publication of a 10-year programme called ‘Strategy for our veterans’ [2] and the 2019 establishment of the Office for Veterans’ Affairs, whose mission is to strengthen lifelong support to veterans [3]. For clarity, the UK government defines a veteran as anyone who has served for 1 day or more [4].

Whilst most veterans successfully reintegrate into civilian life, a significant minority grapple with complex challenges (e.g. [5–9]). These may be complicated by a reluctance to seek assistance associated with internalized mental health stigma, including concerns about being perceived as weak or unworthy of treatment [10]. Furthermore, research highlights organizational barriers, such as limited awareness of where to seek the correct help, inadequate recognition of complex post-traumatic stress disorder (PTSD) symptoms by general practitioners and prolonged waiting times due to overburdened services [10].

PTSD Resolution (PTSD-R [11]) is a UK-based charity that provides treatment for military veterans, reservists and their families who are struggling with military-related PTSD and other mental health conditions. The charity, established in 2009, operates across the UK utilizing a network of over 200 accredited Human Givens Therapy (HGT) therapists [11]. HGT is a collaborative, time-limited, 1:1 therapy providing a holistic approach to mental health treatment grounded in an understanding of the fundamental emotional needs of individuals, empowering them to take an active role in their recovery and helping to develop new coping skills and strategies [12].

A 2019 King’s College London (KCL) evaluation of PTSD-R [13] concluded that PTSD-R appeared to be an acceptable alternative to National Health Service (NHS) treatment through the Improving Access to Psychological Therapies programme (IAPT) [14]. However, the evaluation highlighted that a comparison of HGT and IAPT was challenging because of the use of different measures. Gathering better quality evidence was advised [13].

Whilst Randomized Controlled Trials (RCTs) are ideal, the National Institute for Health and Care Excellence (NICE) recognizes the challenge for organizations such as PTSD-R conducting RCTs and suggests that Real‐World Evidence (RWE) approaches provide useful alternative routes of collecting data [15]. RWE refers to evidence that is collected from routine clinical practice including data from a wide range of patients with varying characteristics, co-morbidities and treatment histories [15]. RWE provides a high level of external validity reflecting the outcomes of interventions in real‐world settings thereby providing insights into the effectiveness of interventions in practice [15].

Therefore, this paper reports on a service evaluation of the therapy provided by PTSD-R building the prior KCL evaluation [13]. Specifically, it sought to establish (i) whether treatment by HGT therapists resulted in positive outcomes at the end of therapy and at follow-up; (ii) factors associated with positive treatment outcomes and (iii) to compare PTSD-R with NHS IAPT data.

## Methods

This service evaluation examined anonymized data of closed cases (i.e. ended therapy or dropped out) collected by PTSD-R therapists between April 2022 and May 2023, therefore drawing from the RWE approach [15]. Raw data of all available closed cases were provided to KCL for independent analysis. For additional clarity, the data extraction and the handover of data to the KCL team were carried out by an individual independent of PTSD-R. PTSD-R clients are routinely asked standard socio-demographic questions including sex, age, ethnicity, employment status, living arrangements (e.g. alone, with partner) and number of dependents. Information on medication use, and disabilities, is also requested. PTSD-R clients complete a range of brief mental health measures when attending sessions. Follow-up data were collected where possible at 3, 6 or 12 months following completion.

IAPT data were extracted from the 2021–22 Annual Report Dashboard and veteran-specific data were identified where possible (available as Supplementary data at *Occupational Medicine* Online, including methods of comparison taken for combined IAPT measures [16]). We used the following IAPT definitions:

*Case or Caseness:* Clients are a ‘case’ when the score is at or above the clinical cut-off for the measure [17].

*Reliable improvement:* Calculated by comparing a client’s pre- and post-treatment scores on an outcome measure and taking into account the reliability of the measure [17].

*Recovery:* Recovered individuals have a final post-treatment score below the given measures’ cut-off [17].

*Reliable recovery:* This is when the final post-treatment score is below cut-off for caseness, and the client has experienced ‘reliable’ positive change [17].

*Depression:* The nine-item Patient Health Questionnaire (PHQ-9), administered before every appointment, measures depression symptoms over the past 2 weeks [18] using a 4-point Likert scale ranging from ‘Not at all’ (0) to ‘Nearly every day’ [3, 18]. A score of ≥10 indicates probable moderate depression, with a decrease of 6 or more points indicating reliable improvement [17].

*Anxiety:* The seven-item Generalized Anxiety Disorder (GAD-7) Questionnaire, administered before every appointment, measures probable anxiety disorder symptoms [19] using the same a 4-point Likert scale as the PHQ-9 [19]. A score of ≥8 in the current evaluation was indicative of probable moderate anxiety disorder, with a decrease of four or more points indicating reliable improvement.

*Post-traumatic Stress Disorder:* The 20-item PTSD Checklist (PCL-5) was used to measure probable PTSD [20] uses a Likert scale ranging from ‘Not at all’ (0) to ‘Extremely’ [5] to assess the frequency of symptoms due to someone’s ‘worst event’ in the past month [20]. A score of ≥32 in the current evaluation was indicative of probable PTSD, with a decrease of 10 or more indicating reliable improvement [17]. The PCL-5 was administered before the first, and either before or immediately after the Client’s final appointment.

Missing entry/completion data was handed pragmatically by only adjusting entry and completion scores if a score was provided the session after (for entry) or the session before (for completion or last known therapy session). Where this was impractical (e.g. missing both the start and second session value), the client was not included in the analysis.

All data management and analyses were performed using IMB SPSS v.27 [21]. First, where necessary variables were recoded to aid analysis (Table 1). Counts, percentages, means and standard deviations (SDs) are presented. The number of cases of probable anxiety, depression and PTSD were computed using score thresholds for both entry and completion scores. Difference in all measure scores between entry and completion was also computed, which allowed indication of average improvement, reliable improvement and reliable recovery. Paired *t-*tests were carried out to investigate if there was a difference between initial and completion scores for each measure; and where possible, investigation into the persistence of a treatment effect using completion and follow-up scores. Univariable binary logistic regressions were used to establish factors associated with providing follow-up data and reliable recovery. Lastly, chi-squared tests were used to establish if there was a significant difference between PTSD-R figures and IAPT for attrition, combined recovery, combined reliable improvement and combined reliable recovery.

**Table 1.** Demographics of 172 PTSD Resolution closed cases

Variable	Count	Percentage
Age		
19–24	3	2
25–34	13	8
35–44	57	33
45–54	53	31
55+	44	26
Gender		
Male	128	74
Female	43	25
Ethnicity		
White	158	92
Other ethnic group	9	5
Employment		
Employed	110	64
Seeking employment	9	5
Not seeking employment	4	2
Sickness	25	15
Retired	18	11
Living arrangement		
Alone	53	31
With partner	89	52
With family	8	5
With non-family	15	9
No fixed abode	4	2
Medication		
Anti-depressant	49	37
Other medication	23	17
None	61	36
Disability		
Yes	29	17
No	53	31

Not all demographics add to the total number of clients due to missing data. All percentages have been rounded to whole numbers.

## Results

A total of 211 closed cases presented to PTSD-R between April 2022 and May 2023 comprising veterans (75%) and their family members (21%; 4% did not provide this information). A minimum of two sessions was attended by 94% (*n* = 198) with a mode of seven sessions per client; 82% (*n* = 173) had a planned ending.

**Figure 1.**
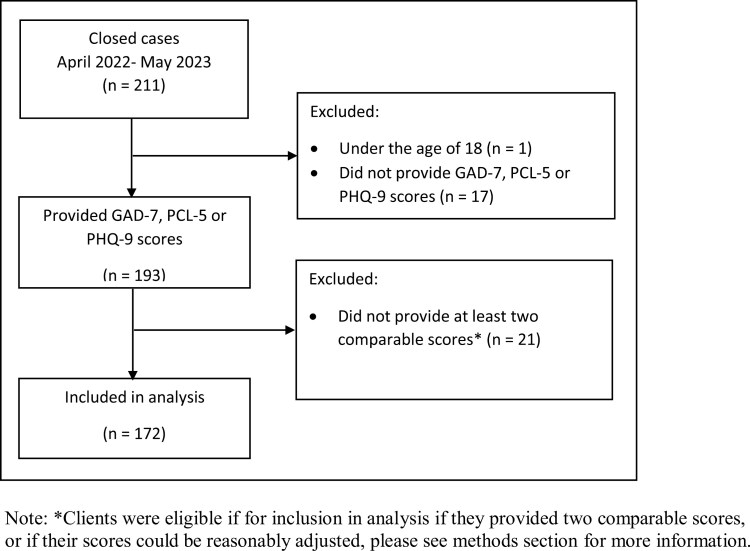
Participant flow diagram.

Of the 211 closed cases, 172 (82%) provided entry and end point GAD-7 [19], PCL-5 [20] or PHQ-9 [18] data scores (see Figure 1). Table 1 presents the demographic distribution of the 172 PTSD-R clients eligible for inclusion in the analysis. In summary, 25% of the ‘included in analysis sample’ were female, age ranged from 18 to 80 (*mean* = 47.92, *SD* = 11.71); just over 90% of clients were White in ethnicity. Clients in this sample attended between three and 20 sessions (mean = 8.00, SD = 3.14).

Of the 172 clients included in the analysis, 90% met the threshold criteria for a probable anxiety disorder, 76% for probable PTSD and 80% for probable depression at their initial assessment. By the final session, 43.2% met the threshold criteria for probable anxiety (decrease by 47%), 29% for probable PTSD (decrease by 47%) and 43% for probable depression (decrease by 37%). Paired *t*-tests indicated a significant difference between the first and last session across all measures (*P* < 0.001). Reliable improvement was apparent for 70% of those with probable anxiety disorder, 72% with probable PTSD and 54% with probable depression. Furthermore, of initial probable cases, consistently around 50% of cases recovered across all three measures (55% anxiety disorder, 66% PTSD, 50.0% depression). Table 2 provides more information.

**Table 2.** IAPT style analysis of GAD-7, PHQ-9 and PLC-5 outcomes

	Probable anxiety (*n* = 169)		Probable PTSD (*n* = 136)		Probable depression (*n* = 166)	
Initial						
Mean (SD)	13.94	(4.97)	45.14	(17.09)	16.18	(6.71)
Probable cases (%)	152	(90)	103	(76)	132	(80)
Completion						
Mean (SD)	7.53	(5.58)	24.69	(18.27)	9.17	(7.05)
Probable cases (%)	73	(43)	39	(23)	71	(43)
Initial versus completion						
Mean difference (SD)	6.41	(5.97)	20.45	(17.86)	7.01	(6.80)
Reliable improvement (%)	118	(70)	98	(72)	90	(54)
Cases recovered (%)	84	(55)	68	(66)	66	(50)
Paired *t*-test	*t*(168) = 13.96 *P* < 0.001		*t*(135) = 13.35 *P* < 0.001		*t*(165) = 13.29 *P* < 0.001	
Initial versus follow-up	(*n* = 48)		(*n* = 42)		(*n *= 48)	
Mean initial (SD)	14.25	(4.62)	45.50	(16.08)	16.58	(6.08)
Mean follow-up (SD)	7.08	(6.18)	27.36	(20.77)	9.44	(7.52)
Paired *t*-test	*t*(47) = 7.48 *P* < 0.001		*t*(41) = 6.36 *P* < 0.001		*t*(47) = 6.65 *P* < 0.001	
Completion versus follow-up	(*n* = 48)		(*n* = 42)		(*n* = 48)	
Mean completion (SD)	5.73	(4.37)	21.26	(16.03)	7.48	(6.01)
Mean follow-up (SD)	7.08	(6.18)	27.36	(20.77)	9.44	(7.52)
Paired *t*-test	*t*(47) = −1.68 *P* = 0.099		*t*(41) = −2.39 *P* = 0.022		*t*(47) = −2.31 *P* = 0.025	

All percentages have been rounded to whole numbers.

Univariable binary logistic regressions established that clients were more likely to make reliable improvement (in combined probable anxiety and depression measures) if they attended more sessions. No significant predictors were found to predict reliable improvement in probable PTSD (Table 3).

**Table 3.** Factors associated with reliable improvement, split by PCL and combined PHQ-9 and GAD-7 measures

		Reliable improvement (PCL)									
Factor	Category	Yes				No				Odds ratio (95% CI)	*P-*Value
		Count	Percentage	Mean	SD	Count	Percentage	Mean	SD		
Sex	Male	73	75%			24	25%			0.59 (0.26–1.31)	0.192
	Female	25	64%			14	36%			Reference	
Age				47.25	11.03			45.97	12.60	0.99 (0.96–1.02	0.558
Ethnicity	White	89	72%			35	28%			0.66 (0.15–2.89)	0.577
	Other ethnic group	5	63%			3	38%			Reference	
Medication	Anti-depressant	29	73%			11	28%			0.76 (0.30–1.92)	0.561
	Other medication	17	90%			2	11%			0.24 (0.05–1.16)	0.075
	None	30	67%			15	33%			Reference	
Disability	Yes	17	68%			8	32%			1.67 (0.55–5.13)	0.367
	No	32	78%			9	22%			Reference	
Planned end	Yes	95	73%			35	27%			0.37 (0.07–1.91)	0.235
	No	3	50%			3	50%			Reference	
Number of sessions				8.83	3.19			7.74	2.88	0.88 (0.77–1.01)	0.072
											
		**Reliable improvement (combined GAD-7 and PHQ-9)**									
Sex	Male	98	82%			22	18%			0.53 (0.23–1.23)	0.141
	Female	26	70%			11	30%			Reference	
Age				48.81	12.20			47.42	10.91	0.99 (0.96–1.02)	0.555
Ethnicity	White	114	79%			31	21%			2.18 (0.26 – 18.01)	0.472
	Other ethnic group	8	89%			1	11%			Reference	
Medication	Anti-depressant	36	78%			10	22%			0.88 (0.34 – 2.24)	0.782
	Other medication	21	96%			1	5%			0.15 (0.02–1.23)	0.077
	None	41	76%			13	24%			Reference	
Disability	Yes	24	86%			4	14%			1.42 (0.39 – 5.13)	0.592
	No	38	81%			9	19%			Reference	
Planned end	Yes	116	82%			26	18%			**0.29 (0.10 – 0.85)**	**0.023**
	No	9	56%			7	44%			Reference	
Number of sessions				8.30	3.16			7.39	3.36	0.91 (0.80 – 1.04)	0.154

All percentages have been rounded to whole numbers.

Follow-up data were provided by 28% (GAD-7), 24% (PCL-5) and 28% (PHQ-9) of clients; the majority of follow-up data was collected after 3 months. Table 2 shows the results of paired *t*-tests comparing completion versus follow-up scores to assess the longevity of treatment. All measures increased between completion and follow-up; significant differences were found for probable PTSD (increase of ~7 points) and probable depression (increase of ~2 points). No significant differences were found for probable anxiety (increase of ~1 point). However, all follow-up scores remained under the threshold for caseness (PCL-5: 27.4; PHQ-9: 9.4 and GAD-7: 7.1). Paired *t*-tests of initial versus follow-up scores (Table 2) showed that all follow-up scores remaining significantly lower than initial score.

Univariate binary logistic regressions showed that those who provided follow-up data were more likely to meet caseness for probable anxiety and probable PTSD at completion of therapy, as well as being more likely to have attended less sessions (Table 4).

**Table 4.** Factors associated with response to follow-up

		Follow-up									
Factor	Category	Yes				No				Odds ratio (95% CI)	*P*-value
		Count	Percentage	Mean	SD	Count	Percentage	Mean	SD		
Sex	Male	38	30%			90	70%			0.92 (0.43–1.97)	0.824
	Female	12	28%			31	72%			Reference	
Age	Age			49.78	10.93			47.18	11.98	0.98 (0.95–1.01)	0.190
Ethnicity	White	44	28%			114	72%			2.07 (0.53 -8.08)	0.294
	Other ethnic group	4	44%			5	56%			Reference	
Employment	Employed	29	26%			81	74%			1.07 (0.35–3.28)	0.900
	Seeking employment	3	33%			6	67%			0.39 (0.04–3.52)	0.398
	Not seeking employment	2	50%			2	50%			0.68 (0.18–2.55)	0.571
	Sickness	9	36%			16	64%			0.77 (0.14–4.33)	0.766
	Retired	5	28%			13	72%			Reference	
Medication	Anti-depressant	14	29%			35	71%			0.75 (0.32–1.76)	0.502
	Other medication	3	13%			20	87%			1.99 (0.51–7.68)	0.320
	None	14	23%			47	77%			Reference	
Disability	Yes	2	7%			27	93%			2.76 (0.56–13.75)	0.215
	No	9	17%			44	83%			Reference	
Planned end	Yes	49	31%			107	69%			0.15 (0.02–1.13)	0.066
	No	1	6%			15	94%			Reference	
Number of sessions				9.46	2.85			7.40	3.08	**0.81 (0.73–0.91)**	**<0.001**
Initial GAD	Yes	46	30%			106	70%			0.71 (0.22–2.29)	0.566
	No	4	24%			13	77%			Reference	
End GAD	Yes	13	18%			60	82%			**2.89 (1.40–5.99)**	**0.004**
	No	37	39%			59	62%			Reference	
Initial PCL	Yes	34	33%			69	67%			0.65 (0.27–1.59)	0.345
	No	8	24%			25	76%			Reference	
End PCL	Yes	7	18%			32	82%			**2.58 (1.03–6.46)**	**0.043**
	No	35	36%			62	64%			Reference	
Initial PHQ	Yes	41	31%			91	69%			0.58 (0.23–1.43)	0.243
	No	7	21%			27	79%			Reference	
End PHQ	Yes	16	23%			55	78%			1.78 (0.87–3.52)	0.119
	No	32	34%			63	66%			Reference	

All percentages have been rounded to whole numbers.

Chi-squared analyses of PTSD-R deemed to have ‘finished treatment’ under IAPT guidelines (i.e. have attended two or more sessions, where there is more than one measurement point available [17]) showed that more PTSD-R, than IAPT, clients finished treatment (i.e. 82% versus 54%; 82% versus 56%; 82% versus 57%) (Table 5).

**Table 5.** IAPT combination data for comparison

	IAPT (England) 2021–2022	IAPT (England Veterans) 2021–2022	IAPT (Pennine Care Veterans) 2021–2022	PTSD Resolution 2022–2023
Number that accessed services	1.24 million	19,800	270	211
Clients finishing treatment^a^	54%	56%	57%	82%
Chi-squared comparison	χ^2^(1) = 66.31, *P* < 0.000	χ^2^(1) = 54.41, *P* < 0.000	χ^2^(1) = 31.63, *P* < 0.000	Comparison
Recovery rate	50%	53%	49%	47%
Chi-squared comparison	χ^2^(1) = 0.67, *P* = 0.413	χ^2^(1) = 2.31, *P* = 0.129	χ^2^(1) = 0.14, *P* = 0.708	Comparison
Reliable improvement rate	67%	68%	76%	79%
Chi-squared comparison	χ^2^(1) = 10.91, *P* = 0.001	χ^2^(1) = 8.49, *P* = 0.004	χ^2^(1) = 0.50, *P* = 0.482	Comparison
Reliable recovery rate	47%	50%	48%	46%
Chi-squared comparison	χ^2^(1) = 0.14, *P* = 0.711	χ^2^(1) = 1.26, *P* = 0.262	χ^2^(1) = 0.22, *P* = 0.643	Comparison

Data were extracted from the 2021–22 Dashboard for the Annual report on the use of IAPT services in England [16]. Veteran-specific data have been used where possible (i.e. extracted from Page 25—Ex-British armed forces and dependents outcome measure, and refined by provider to access Pennine Care NHS Foundation Trust outcomes) [16]. Where possible, analyses used specific numbers available from the IAPT sources (i.e. in the case of referrals finishing treatment), where this was not possible, given percentages were used to work out estimates for comparison. All percentages have been rounded to whole numbers.

^a^Clients were deemed to have finished treatment if attended two or more sessions, where there is more than one measurement point available [17].

Of the 172 clients in the included in analysis sample, 170 completed either, or both, the GAD-7 and PHQ-9 at their initial assessment. A total of 91% (*n* = 156) of these could be classified as a combined case (i.e. met caseness for probable anxiety or depression). By the final session, 47% had recovered, 79% had reliably improved and 46% had reliably recovered.

Chi-squared was used to compare percentages of recovery, reliable recovery and reliable improvement between PTSD-R (sample of 170 closed cases) and each of the three sources. In summary, no significant differences were found between all three IAPT data sources and PTSD-R in terms of recovery or reliable recovery. Significant differences were found between PTSD-R figures and two of the IAPT data sources for reliable improvement, with PTSD-R showing a higher percentage of reliable improvement (i.e. 79% versus 67%; 79% versus 68%), more information can be found in Table 5.

## Discussion

Across a sample of 172 closed cases who enrolled in therapy with PTSD-R between April 2022 and May 2023, 90% met the threshold criteria for a probable anxiety disorder, 76% for probable PTSD and 80% for probable depression at their initial assessment. By the final session, scores had significantly decreased across all mental health measures. Although there were small but significant, increases in PTSD and depression scores by the 3-month follow-up point, mean scores for all measures remained below the threshold for probable caseness. We found that PTSD-R patients did not significantly differ from any extracted IAPT figures for recovery, or reliable recovery rates [16]. PTSD-R also did not differ from Pennine Care Veterans IAPT services in terms of reliable improvement (i.e. 79% versus 76% [16]), but did significantly differ from England level (i.e. 79% versus 67% [16]), and Veteran level IAPT figures (i.e. 79% versus 68% [16]); more specific information can be found in Table 5. Additionally, those with a planned end were likely to experience positive reliable improvement.

We found that the current sample of PTSD-R clients was broadly representative of veterans in terms of ethnic origin (92% versus 96% [22]), but differed in gender, age and employment status. A total of 25% of clients were female, which is increased in comparison to UK veteran demographics (10% and 14% [22, 23]), and in veteran mental health literature (e.g. 92% male, 92% male [24, 25]). The most common age group in PTSD-R clients was between 35 and 44, which is in line with characteristics of UK veterans presenting to a psychological therapies service in 2011–2013 (mean of 43 [25]), whereas UK veteran demographics suggest that over half of veterans are over the age of 65, with the most common age group being 80–84 [22]. Furthermore, 47% of UK veterans reported to be employed, and a further 46% retired [23]. Whereas PTSD-R clients have a higher percentage of those in employment (64%) and a lower percentage of retired (11%).

The current paper builds on the KCL service evaluation of PTSD-R in 2019 [13]. The results are complementary in that PTSD-R clients appeared to show a similar degree of improvement as IAPT patients, suggesting consistent results over time. The current evaluation provides further insight into follow-up outcomes, as well as side-by-side comparisons of IAPT data across three levels.

Follow-up data were provided by around 25% of clients, with the majority being collected after 3 months. Results indicated that scores marginally increased after the end of therapy (i.e. end of therapy versus follow-up). Wider research normalizes this finding. For example, 12% of a sample undergoing exposure therapy to aid obsessive–compulsive disorder, experienced a formal relapse at 3-month follow-up after therapy cessation [26]. However, in the current evaluation, the scores observed in the current service evaluation at follow-up were significantly lower than initial scores (i.e. start of therapy versus follow-up). This indicates that the positive mental health benefit of engaging with a PTSD-R therapist was sustained. This is particularly showcased by significant regression results which indicate that participants who had engaged in less sessions and were more likely to remain above caseness threshold for probable anxiety and depression measures at completion, were more likely to provide follow-up data. When considering these caveats, it suggests that if follow-up data were obtained from all PTSD-R clients in the sample, the follow-up outcomes would be more positive.

We found that of the sample of 211 closed cases, only around 6% of clients attended only one session. On average, clients attended a mode of seven sessions, with 82% having a planned ending. Using IAPT guidelines (i.e. have attended two or more sessions, where there is more than one measurement point available [17]), 82% of PTSD-R clients were deemed to have ‘finished treatment’, which was a significantly higher rate in comparison to all extracted IAPT figures which were all consistently around 50% (Table 5). We also established that PTSD-R clients recovered, reliably improved and reliably recovered in line with IAPT patients when looking at combined probable anxiety and depression outcomes. NHS England reports that for NHS Talking Therapies (for anxiety and depression) at least half of people who complete a course (i.e. attend two or more sessions) of treatment should recover, and around two-thirds should reliably improve [27]. The current service evaluation found PTSD-R to have a recovery rate and reliable improvement rate in line with NHS England standards (47% and 79%, respectively).

In terms of limitations, first, the current paper seeks to evaluate existing services. Ideally, such a study would utilize an RCT methodology, but this approach is costly and complex. Instead, we used a Real-World Evidence Framework approach [15] which was both practical and in keeping with NICE given the context (i.e. it would unethical or unfeasible to require an RCT due to unmet need and funding constraints of the charity [28]). Second, in relation to missing data, a pragmatic approach was taken by the research team in that entry/completion scores were only adjusted if considered practical (i.e. were taken the following, or previous session). As the time between sessions would have been minimal (i.e. around a week), it is infeasible that those seeking mental health support would have recovered following one session. However, we acknowledge that this is a limitation and should be considered when interpreting these results. Third, follow-up data were only provided by around a quarter of the sample and varied on time scale. For example, follow-up could have been made at the 3-, 6- or 12-month point. However, examination of the data suggests that the majority of follow-up data was collected 3 months post the final session, meaning that the effect should not be assumed to persist. Furthermore, IAPT does not routinely collect follow-up data, therefore limiting comparisons. Fourth, only basic information about medication use was available with potentially relevant detail (e.g. change to dosage) not being reported which could act as an influential external factor in change to symptoms. Lastly, the sample size used within this service evaluation is relatively small, particularly regarding the follow-up data. However, this was determined by the number of closed cases meeting the inclusion criteria between April 2022 and May 2023 (and those who decided to complete follow-up). Clients are also from a select group (i.e. veterans), making broader comparisons difficult. Thus, this should be considered when interpreting these results, and future service evaluations in this area should seek to include larger amounts of participants where possible.

The results of this service evaluation suggest that veterans who enter therapy with PTSD-R appear to experience similar rates of recovery to IAPT users. The vast majority of those starting in treatment completed it and reached a planned ending (82%); those who reached a planned end with PTSD-R were more likely to experience positive outcomes. Analysis of follow-up data (collected for 25% of clients) revealed that clients symptom scores slightly increased following the completion of therapy but, in the main, remained below caseness thresholds and significantly lower than entry-level scores. These data suggest that veterans who choose to engage with PTSD-R for their mental health difficulties should expect to experience a similar benefit to that they would have experienced if they had sought outpatient care from the NHS.

## Data Availability

The dataset used and/or analysed during the current study is available from the corresponding author upon reasonable request.
